# The Tumorigenicity of Multipotent Adult Germline Stem Cells Transplanted into the Heart Is Affected by Natural Killer Cells and by Cyclosporine A Independent of Its Immunosuppressive Effects

**DOI:** 10.3389/fimmu.2017.00067

**Published:** 2017-02-06

**Authors:** Daniela Hübscher, Diana Kaiser, Leslie Elsner, Sebastian Monecke, Ralf Dressel, Kaomei Guan

**Affiliations:** ^1^Department of Cardiology and Pneumology, University Medical Center Göttingen, Göttingen, Germany; ^2^DZHK (German Center for Cardiovascular Research), Partner Site Göttingen, Göttingen, Germany; ^3^Institute of Cellular and Molecular Immunology, University Medical Center Göttingen, Göttingen, Germany; ^4^Institute of Pharmacology and Toxicology, Technische Universität Dresden, Dresden, Germany

**Keywords:** tumorigenicity, multipotent adult germline stem cells, microenvironment of the heart, natural killer cells, cyclosporine A, cell transplantation

## Abstract

Transplantation of stem cells represents an upcoming therapy for many degenerative diseases. For clinical use, transplantation of pluripotent stem cell-derived cells should lead to integration of functional grafts without immune rejection or teratoma formation. Our previous studies showed that the risk of teratoma formation is highly influenced by the immune system of the recipients. In this study, we have observed a higher teratoma formation rate when undifferentiated so-called multipotent adult germline stem cells (maGSCs) were transplanted into the heart of T, B, and natural killer (NK) cell-deficient RAG2^−/−^γc^−/−^ mice than in RAG2^−/−^ mice, which still have NK cells. Notably, in both strains, the teratoma formation rate was significantly reduced by the immunosuppressive drug cyclosporine A (CsA). Thus, CsA had a profound effect on teratoma formation independent of its immunosuppressive effects. The transplantation into RAG2^−/−^ mice led to an activation of NK cells, which reached the maximum 14 days after transplantation and was not affected by CsA. The *in vivo*-activated NK cells efficiently killed YAC-1 and also maGSC target cells. This NK cell activation was confirmed in C57BL/6 wild-type mice whether treated with CsA or not. Sham operations in wild-type mice indicated that the inflammatory response to open heart surgery rather than the transplantation of maGSCs activated the NK cell system. An activation of NK cells during the transplantation of stem cell-derived *in vitro* differentiated grafts might be clinically beneficial by reducing the risk of teratoma formation by residual pluripotent cells.

## Introduction

Pluripotent stem cell (PSC)-based cellular transplantation represents an upcoming therapy for many degenerative diseases such as heart failure (1, 2). Embryonic stem cells (ESCs) are pluripotent and are able to differentiate into derivatives of the three embryonic germ layers. Therefore, they could be used to obtain a broad variety of cellular grafts including cardiomyocytes or cardiac tissues for cellular therapies (3). However, the establishment of human ESC-based cellular therapies causes considerable ethical concerns due to the need to destroy blastocysts for generating ESCs. In the past decade, many efforts have been made to generate induced pluripotent stem cells (iPSCs) by genetically manipulating somatic cells (4–6). The iPSCs overcome the ethical concerns associated with ESCs. We have shown that mouse adult spermatogonial stem cells (SSCs) are able to be converted into so-called multipotent adult germline stem cells (maGSCs), which exhibit similar differentiation properties as ESCs, by modifying the culture conditions of the SSCs without the need of any genetic manipulation (7). These PSCs provide us the opportunity to study on an alternative PSC-based cellular therapy for the future clinical application (8).

For clinical use of PSCs, one of the critical issues is the risk of teratoma formation after transplantation of desired differentiated cells that might contain traces of PSCs (9). Generally, the risk of teratoma formation associated with PSCs is due to their proliferation and differentiation capacity. It is widely debated whether the procedures used for the generation of iPSCs further increase the risk of tumor formation (10, 11). For allogeneic and perhaps even for syngeneic transplantations (due to the expression of developmental antigens or neo-antigens) of PSC-derived grafts, another issue is immune rejection of the transplanted cells (12). Although terminally differentiated PSC-derived syngeneic cells are apparently accepted in recipients (13–15), the immunogenicity of therapeutically relevant autologous grafts was reported to differ depending on the cell types into which human iPSCs have been differentiated (16).

Early studies showed that injection of undifferentiated murine ESCs, iPSCs, or maGSCs into immunodeficient or syngeneic mice led to teratoma formation, demonstrating engraftment (7, 17–22). When mouse or human ESCs or iPSCs were injected into immunocompetent allogeneic or xenogeneic mice, respectively, they were rejected by the immune system and no teratoma formation was observed (20, 23, 24). In a previous study, we observed a failure of engraftment after allogeneic transplantation of ESCs subcutaneously in immunocompetent mice (0 out of 32 mice) and a rare engraftment, i.e., tumor formation (1 out of 27 mice) in mice treated with cyclosporine A (CsA) (20). CsA, a calcineurin inhibitor, is one of the most commonly used immunosuppressive drugs after solid organ transplantation. It inhibits the production of interleukin (IL)-2, thus suppressing the activation of alloreactive T lymphocytes (25). Notably, after allogeneic transplantation of mouse ESCs into the heart, teratomas were observed even in immunocompetent mice not treated with CsA or other immunosuppressive drugs (18). However, when we injected maGSCs into the heart of immunocompetent allogeneic mice immunosuppressed daily with CsA, no teratomas were found within 4 weeks (8). These data suggest that besides the immune system of the recipients, the source, quality, and differentiation state of the injected cells exert huge influence on the transplantation outcome and specifically on the risk of teratoma formation.

Different elements of the immune system could be responsible for the rejection of transplanted PSCs. In addition to T lymphocytes (23, 26), natural killer (NK) cells play an important role in the rejection of stem cell grafts (20–22, 27, 28). Our previous studies showed that mouse PSCs, including ESCs, iPSCs, and maGSCs, are targets for allogeneic and syngeneic IL-2-activated NK cells *in vitro* (20, 22). Moreover, NK cells impair the growth of teratomas after subcutaneous injection of PSCs, including ESCs, iPSCs, and maGSCs (20, 22, 29). Similarly, human iPSCs are targets for allogeneic and syngeneic IL-2-activated NK cells (30). NK cells are often described as first-line defense against infected or malignant cells, which act without the need of prior activation. However, the regulation of NK cell activity is now known to be much more complex (31).

Natural killer cells might be important after transplantation of grafts derived from PSCs because they could have the ability to reject residual PSCs, which might contaminate grafts in very low numbers, despite all efforts to eliminate undifferentiated cells by various means (29). Differentiated cells, in contrast, are expected to have the ability to inhibit NK cells and should therefore not become a target of these cytotoxic cells (22). However, so far, it is not known whether transplantation of allogeneic PSCs can activate NK cells in recipients receiving a treatment, e.g., with CsA, to suppress the immune system.

In the present study, we show that the NK cell cytotoxicity was increased after transplantation of maGSCs into the hearts of B and T cell-deficient, immunocompetent, and CsA-treated immunosuppressed recipients. The cardiac operation procedure alone was sufficient to activate NK cells against the PSCs. Moreover, NK cells reduced the frequency of teratoma formation after transplantation of maGSCs into the heart. Notably, CsA had an independent effect on the maGSCs, which further reduced the risk of teratoma formation in the recipients.

## Materials and Methods

### Cell Culture

Mouse maGSCs (line SSC5) were cultured on mitomycin C-inactivated mouse embryonic feeder cells in Dulbecco’s modified eagle medium (DMEM; Thermo Fisher Scientific) supplemented with 15% fetal calf serum (FCS; selected batch, Lonza), glutamine (2 mM, Thermo Fisher Scientific), 1× non-essential amino acids (Thermo Fisher Scientific), β-mercaptoethanol (β-ME; 50 µM, Promega), and 10^3^ U/ml leukemia inhibitor factor (Millipore) as described previously (7). The cells have been derived from a mouse with a mixed FVB (H2^q^), C57BL/6 (H2^b^), and 129Sv (H2^b^) genetic background (7). For *in vivo* studies, the maGSCs were separated from the mouse embryonic feeder cells before use. The maGSCs were obtained by collecting the floating cells after being cultivated for 1 h on culture dishes coated with 0.1% gelatin (Sigma-Aldrich: Fluka).

The murine T-lymphoma cell lines YAC-1 (H2^a^), which was used as positive control for the cytotoxic activity of NK cells, and RMA (H2^b^) were maintained in DMEM supplemented with 10% FCS, 2 mM glutamine, 1 mM sodium pyruvate, 50 µM β-ME, 100 U/ml penicillin, and 100 µg/ml streptomycin.

### Intramyocardial Injection of maGSCs

Following mice strains were used in the study: C57BL/6 wild-type mice, immunodeficient RAG2^−/−^ mice that have no B and T lymphocytes, and RAG2^−/−^γc^−/−^ mice that are deficient of B and T lymphocytes and NK cells. All animal experiments were carried out in compliance with EU legislation (Directive 2010/63/EU). The animal experiments for transplantation of maGSCs into the heart were approved by Lower Saxony State Office for Animal Welfare (Az: 33.425.2-010/06) and for subcutaneous injection of maGSCs by Lower Saxony State Office for Consumer Protection and Food Safety (Az: 33.14.42502-04-113/09).

Mouse maGSC SSC5 cells were injected in the anterolateral wall of the heart as described previously (8). Briefly, mice were anesthetized with isoflurane, intubated with a 22 gauge (G) plastic cannula and ventilated with a mixture of 2% isoflurane in ambient air (150 breaths/min, tidal volume 150 µl) by use of a MiniVent (Type 845, Harvard Apparatus). The heart was exposed by a left lateral thoracotomy via the fourth intercostal space and the pericardium was opened. Undifferentiated maGSCs (3 × 10^5^) were injected in a volume of 20 µl by use of a 30G steel cannula connected to a 50 µl Hamilton syringe via a PE10 tube. The total volume was applied in four injections of 5 µl each covering the area of the anterolateral wall of the heart that was accessible via the thoracotomy. Afterward, the thorax was closed with single sutures before the skin was adapted with polypropylene 6-0 sutures. After surgery, the mice received buprenorphine (0.1 mg/kg body weight, intraperitoneal injection) for pain relief and were placed on a heating pad to recover until fully awake. Metamizole (1.33 mg/ml) was provided with the drinking water to the mice for postoperative analgesia for a week.

The mice were sacrificed at different time points, i.e., 3, 7, 14, or 28 days after the cell transplantation (Tx) and the hearts and spleens were taken out for histological or immunological analyses, respectively. Blood was collected to obtain serum that was stored at −20°C until used for cytokine measurements. The mice were inspected for tumors at other sites except for the heart. If present, such tumors were taken out for further histological analyses.

### Histological Studies

The hearts and tumors found at other sites of the body were fixed in phosphate-buffered formalin (30 mM NaH_2_PO_4_, 40 mM Na_2_HPO_4_, 4% formalin) for 4 h at room temperature. The tissues were then washed three times with distilled water, embedded in paraffin, and cut into 6 µm thin sections, which were examined with hematoxylin and eosin (H&E) staining on an automated immunostainer (Ventana Medical Systems) according to the manufacturer’s protocol.

For immunohistological analysis, deparaffinized sections were boiled in citrate buffer (DakoCytomation) for 15 min to recover the antigenicity. Afterward, the sections were incubated in 3% hydrogen peroxide for 10 min at room temperature to block endogenous peroxidases and in 4% bovine serum albumin (Thermo Fisher Scientific) for 30 min to block non-specific immunoreactivity. The sections were then treated with 0.2% Triton X-100 (Sigma) for 30 min before incubation with the primary antibody (anti-OCT4 monoclonal antibody, BD Biosciences, dilution 1:100) for 60 min at room temperature. OCT4 was then detected with commercial available SuperPicture™ Polymer Detection Kit (Thermo Fisher Scientific).

### Isolation of NK Cells

For the analysis of NK cell cytotoxicity, the spleens were taken out and the NK cells were isolated by the magnetic-activated cell sorting using a kit (Miltenyi Biotec) to obtain untouched mouse NK cells by negative selection according to the manufacturer’s instruction. The purity of the isolated CD49b^+^CD3^−^ NK cells was always determined by flow cytometry.

### ^51^Chromium Release Assay

Target cells were labeled by incubating 1 × 10^6^ maGSC SSC5 or YAC-1 cells with 200 µl of DMEM with 100 µl of FCS and 50 μCi Na_2_^51^CrO_4_ (Hartmann Analytic) for 1 h at 37°C and then washed three times with DMEM. These ^51^Chromium-labeled target cells (5 × 10^3^ per well of round-bottomed microtiter plates) were co-cultivated with the isolated NK cells (effector cells) in triplicates at diverse effector–target ratios in 200 µl of DMEM containing 10% FCS for 4 h at 37°C. The microtiter plates were centrifuged for 5 min at 40 *g*; supernatant and sediment were separately taken to determine radioactivity in each well using a Wallac MicroBeta Trilux counter (PerkinElmer Life Sciences). Spontaneous release was determined by incubating target cells without the effector cells. The percentage of specific lysis of the target cells was calculated as described previously (22, 32).

### Flow Cytometry

Flow cytometry was performed with a FACSCalibur flow cytometer and CellQuestPro software (BD Biosciences). The purity of isolated NK cells was analyzed using monoclonal antibodies (mAbs) against CD49b and CD3. The Abs used for flow cytometry are described in Table S1 in Supplementary Material. Cell surface expression of CD112, CD155, RAE-1, H2K^b^, H2D^b^, and H2K^q^ was determined on target cells. In some experiments, interferon (IFN)-γ (100 ng/ml, ImmunoTools) was added to the cells 48 h before analysis. Isotype controls were used for directly labeled mAbs. For staining, 2 × 10^5^ cells were incubated in 100 µl PBS with 1 µg of the respective primary mAb for 30 min at 4°C before washing with PBS. To detect the unlabeled anti-CD112, anti-CD155, and anti-RAE-1 mAbs, the cells were incubated subsequently in 100 µl PBS with 1 µl of FITC-labeled anti-rat IgG Ab for 30 min. Cells stained with the secondary Ab only served as control.

### Enzyme-Linked Immunosorbent Assay (ELISA)

To measure the concentration of IL-1b, IL-4, IL-6, IL-17A, IFN-γ, and tumor necrosis factor (TNF)-α in sera of mice, we used ELISA MAX Standard Sets from Biolegend according to the manufacturer’s instructions. The sera were diluted 1:2 for the measurement of IL-1b and TNF-α and 1:5 for the other cytokines. In the end, 50 µl 2,2′-azino-bis(3-ethylbenzothiazoline-6-sulfonic acid) (ABTS) substrate solution was added to each well and the optical density was determined using a BioTek PowerWave 340 microplate spectrophotometer (BioTek) set to 405 nm. All samples were analyzed in comparison to a standard curve of the respective cytokine.

### Statistical Analysis

Results are shown as mean with SD or as mean with SEM for cytotoxicity assays. The data were evaluated with the GraphPad Prism 4.0 or 6.0 program or WinStat software (R. Fitch Software). The Kolmogorov–Smirnov test was used to determine normal distribution and Student’s *t*-tests or two-way-ANOVAs for subsequent analyses. The non-parametric Wilcoxon (*U*) or Kruskal–Wallis (*H*) tests were used to compare non-normally distributed target variables. Pearson’s chi-squared test (χ^2^) was used to analyze differences in sets of categorical data. A *P*-value of ≤0.05 in two-sided tests was considered significant. Significance levels are shown in the following way, if not the exact *P* value is given: *P ≤ 0.05; **P ≤ 0.01; ***P ≤ 0.001.

## Results

### The Microenvironment of the Heart Does Not Affect the Tumorigenicity of maGSCs

Our previous studies showed that undifferentiated maGSCs (line SSC5) could form teratomas in immunodeficient SCID mice within 6 weeks after subcutaneous injection (2 × 10^6^ cells per injection) (7) and the cells retained this capacity under different culture conditions (21). However, when the SSC5 maGSCs (0.5–1 × 10^6^ cells per injection) were injected into the heart of partially allogeneic C57BL/6 wild-type mice immunosuppressed daily with CsA (10 mg/kg body weight, i.p.), no engraftment and no teratomas were found within 4 weeks (8). Here, three questions were raised: (1) Does the microenvironment of the heart prevent the tumorigenicity of maGSCs? (2) Do NK cells play a role in the rejection of the PSCs in CsA-treated hosts? (3) Does the treatment of the recipients with CsA affect the maGSCs directly?

**Figure 1:**
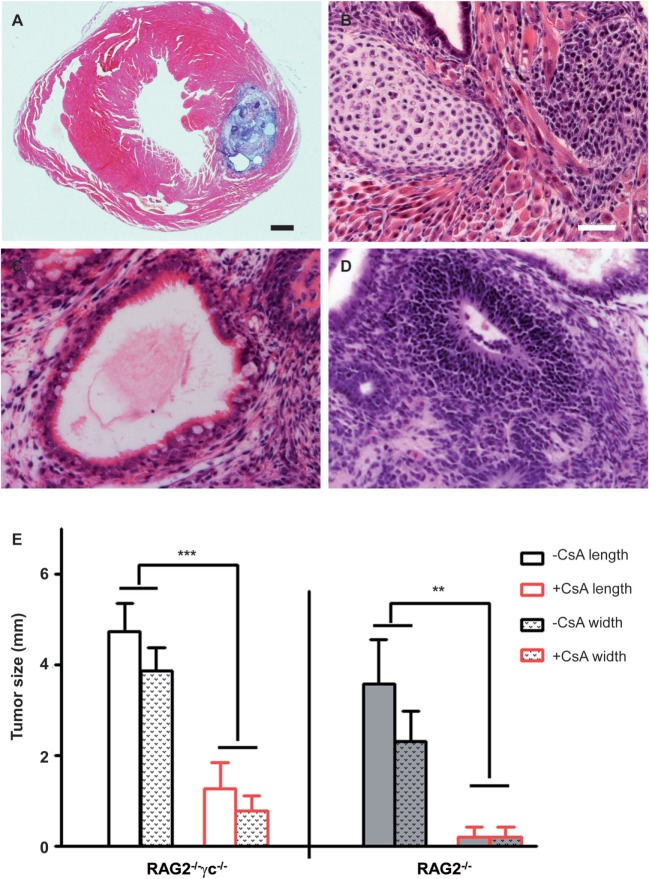
**Teratomas in immunodeficient RAG2^−/−^γc^−/−^ and RAG2^−/−^ mice with and without cyclosporine A (CsA) treatment**. **(A)** H&E staining of an intramyocardial teratoma after injection of undifferentiated multipotent adult germline stem cells (maGSCs) into immunodeficient RAG2^−/−^γc^−/−^ mice. An overview is shown of the teratoma in the myocardium. **(B)** Cartilage and muscle cells indicate a mesodermal differentiation within this teratoma. **(C)** An endodermal differentiation is represented by intestinal epithelium. **(D)** The neural rosette represents an ectodermal differentiation in the teratoma. The scale bar in **(A)** represents 500 µm and in **(B–D)** indicates 50 µm. **(E)** The hearts of mice (RAG2^−/−^γc^−/−^ and RAG2^−/−^) were sectioned and analyzed at day 28 after injection of undifferentiated maGSCs in the heart. The maximal length and width of the tumor section in each heart was measured and mean + SD are shown. The groups RAG2^−/−^γc^−/−^ − CsA (*n* = 8) and RAG2^−/−^γc^−/−^ + CsA (*n* = 6), or RAG2^−/−^ − CsA (*n* = 6) and RAG2^−/−^ + CsA (*n* = 5), have been compared by two-way ANOVA for length and width. The respective *P* values are indicated.

To answer these questions, first we examined whether the microenvironment of the heart influences the tumorigenicity of undifferentiated maGSCs. The maGSC SSC5 cells (5 × 10^5^ cells per injection) were transplanted directly into the myocardium of RAG2^−/−^γc^−/−^ mice that are deficient in T and B lymphocytes as well as NK cells. All mice (*n* = 8) formed tumors in the heart within four weeks after transplantation (Table 1). The tumors contained derivatives of the three embryonic germ layers, including intestinal epithelium (endoderm), striated muscles, cartilage (mesoderm), and neural tissues (ectoderm), and were therefore teratomas (Figures 1A–D). The teratomas showed a mean size of 4.7 mm length and 3.9 mm width on average (Figure 1E). In addition, teratomas were also formed in the kidney, liver, or intestinal tissues in six out of eight mice (Table 1).

**Table 1:** **Frequency of teratoma formation after transplantation of multipotent adult germline stem cell (maGSC) SSC5 cells**.

Recipient strain	Site of transplantation	Number of transplanted cells	−CsA		+CsA		Duration of experiment (weeks)
			Tumor at site of injection	Tumor at distant site	Tumor at site of injection	Tumor at distant site	
RAG2^−/−^γc^−/−^	Heart	5 × 10^5^	100% (8/8)	75% (6/8)	60% (3/5)	0% (0/5)	4
RAG2^−/−^	Heart	5 × 10^5^	83% (5/6)	33% (2/6)	20% (1/5)	0% (0/5)	4
C57BL/6	Heart	5 × 10^5^	0% (0/7)	0% (0/7)	0% (0/6)^a^	0% (0/6)	1–4^b^
C57BL/6	s.c.	2 × 10^6^	0% (0/9)	0% (0/9)	0% (0/9)	0% (0/9)	15

*^a^In our previous study, no tumors were observed after injection of 0.5–1 × 10^6^ maGSC SSC5 cells into the hearts of six C57BL/6 mice treated with cyclosporine A (CsA) (10 mg/kg/day) at 4 weeks after transplantation (8)*.

*^b^In each group, three mice were sacrificed after 1 week and three (+CsA) or four (−CsA) mice after 2 weeks. In addition, three mice in each group were analyzed 3 days after transplantation and no tumors were found. Sham-operated C57BL/6 mice (*n* = 3 for −CsA and *n* = 3 for +CsA, per time point) received PBS instead of maGSCs. As expected, no tumors were observed at days 3, 7, and 14*.

We also performed subcutaneous injections of the maGSC SSC5 cells into C57BL/6 wild-type mice. The cells (2 × 10^6^ cells per injection) failed to form teratomas after subcutaneous injection in immunocompetent (*n* = 9) and in CsA-immunosuppressed (10 mg/kg/day) recipients (*n* = 9) within 15 weeks after transplantation (Table 1). These data indicate that the microenvironment of the heart has no specific influence on the engraftment or tumorigenicity of undifferentiated maGSCs. Teratomas were formed in immunodeficient recipients similarly in the subcutaneous tissue (7) and in the heart (Table 1), but engraftment failed at both sites in immunosuppressed but partially allogeneic hosts (Table 1). Since CsA mainly suppresses T cells, the results suggested an effect of NK cells on engraftment of maGSCs.

### Tumorigenic Cells Are Targets of NK Cells after Transplantation

To investigate whether NK cells indeed have effects on the teratoma growth in the heart as they have after subcutaneous injections (22), we injected 5 × 10^5^ undifferentiated maGSCs into the myocardium of RAG2^−/−^ mice that are deficient in T and B lymphocytes but have functional NK cells. We detected tumors in the heart in five out of six mice 4 weeks after transplantation (Table 1). Compared to the tumors of the RAG2^−/−^γc^−/−^ mice, the size of teratomas in RAG2^−/−^ mice was smaller with a mean size of 3.6 mm length and 2.3 mm width (Figure 1E). In addition, only in two mice, tumors were also found in the kidney or in the chest. The tumors in RAG2^−/−^ mice also contained derivatives of the three embryonic germ layers, including intestinal epithelium, striated muscles, cartilage, and neural tissues, and were therefore teratomas (data not shown). These results indicate that the NK cells in RAG2^−/−^ mice partially suppress the teratoma formation in the heart and at distant sites of the injection.

**Figure 2:**
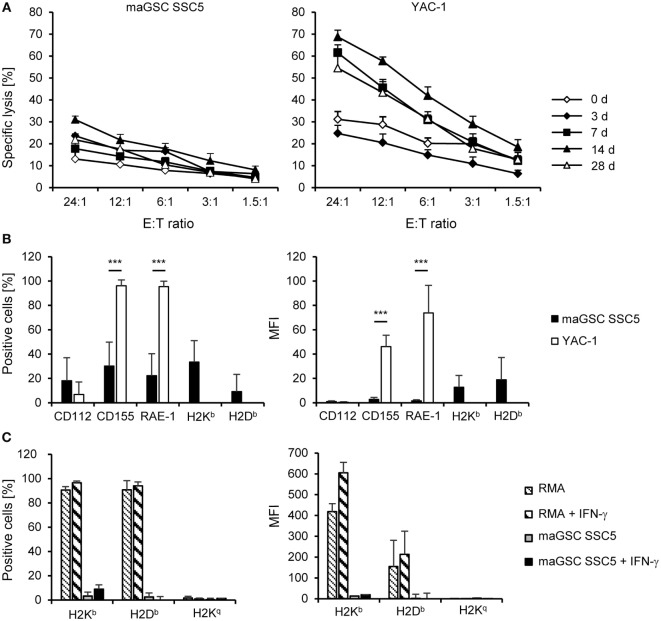
**Cytotoxic activity of natural killer (NK) cells at different time points after intramyocardial transplantation of multipotent adult germline stem cell (maGSC) SSC5 cells in RAG2^−/−^ mice**. **(A)** A summary of means of specific lysis and the SEM is shown as determined by ^51^Cr-release assays of maGSC SSC5 (left panel) and YAC-1 target cells (right panel) by NK cells. The effector:target (E:T) ratio indicates the ratio of CD49^+^CD3^-^ NK cells to maGSC SSC5 or YAC-1 cells. The NK cells were isolated from RAG2^−/−^ mice either before (0 days, *n* = 4) or at various days (3, 7, 14, or 28, *n* = 3 for each group) after intramyocardial transplantation of maGSC SSC5 cells and directly assayed without prior stimulation *in vitro*. **(B)** A flow cytometric analysis of ligands for activating and inhibitory NK receptors on maGSC SSC5 and YAC-1 cells was performed in parallel to the ^51^Cr-release assays. A summary (mean + SD, *n* = 9) is displayed of the percentage of cells expressing the indicated molecules (left panel) and their expression intensity [mean fluorescence intensity (MFI), right panel]. **(C)** The expression of major histocompatibility class I molecules was determined also after stimulation with interferon (IFN)-γ (100 ng/ml for 48 h) by flow cytometry. In these experiments, H2K^q^ was included since the cells have a mixed H2^b^ and H2^q^ background. RMA cells (H2^b^) served as control for the IFN-γ treatment. A summary (mean + SD, *n* = 3) is displayed of the percentage of cells expressing the indicated molecules (left panel) and their expression intensity (MFI, right panel).

We isolated NK cells from the spleens of the RAG2^−/−^ mice (*n* = 3 per group) at different time points after transplantation of the maGSCs (days 3, 7, 14, and 28) and directly analyzed their cytotoxic activity against the SSC5 maGSCs and the NK target cell line YAC-1 in ^51^Chromium release assays. Mice not operated (day 0, *n* = 4) were used to determine the basal NK cell activity. The specific lysis of YAC-1 cells by NK cells of untreated mice was 31% at an effector:target (E:T) ratio of 24:1, but only 13% for the maGSC SSC5 cells (Figure 2A). Thus, the maGSC SSC5 cells were more resistant to NK cells from control mice than the YAC-1 cells (*P* = 7.50 × 10^−8^, two-way ANOVA adjusted for E:T ratios). Also NK cells from transplanted mice killed more YAC-1 than maGSC SSC5 cells (overall: *P* = 7.44 × 10^−17^; 7 days: *P* = 1.23 × 10^−10^; 14 days: *P* = 1.11 × 10^−11^; 28 days: *P* = 4.41 × 10^−8^) with the exception of the earliest time point after transplantation (3 days: *P* = 0.3709, two-way ANOVA adjusted for E:T ratios).

With exception of the earliest time point (3 days), the cytotoxic activity of the NK cells after transplantation of maGSC SSC5 cells into the heart was increased in comparison to those from non-operated control mice (day 0). On average, the specific lysis of YAC-1 cells at an E:T ratio of 24:1 was 62% by NK cells from mice at day 7 (*P* = 0.0003 versus control mice, two-way ANOVA adjusted for E:T ratios), 69% at day 14 (*P* = 2.08 × 10^−8^), and 55% at day 28 after transplantation (*P* = 0.0063). Three days after transplantation, the cytotoxic activity of NK cells against YAC-1 cells was reduced compared to control mice (*P* = 0.0015, two-way ANOVA adjusted for E:T ratios) and the specific lysis was 25% at an E:T ratio of 24:1. In contrast, the specific lysis of maGSC SSC5 cells was increased 3 days (24% at an E:T ratio of 24:1, *P* = 0.0016) but not 7 days after transplantation (18% at an E:T of 24:1, *P* = 0.1446, two-way ANOVA adjusted for E:T ratios). However, 14 days (31% at an E:T ratio of 24:1, *P* = 1.42 × 10^−5^) and 28 days after transplantation (22% at an E:T ratio of 24:1, *P* = 0.0440, two-way ANOVA adjusted for E:T ratios) the specific lysis of maGSC SSC5 cells was also higher than before transplantation. Thus, at day 14 after transplantation, the maximal activity of NK cells against both YAC-1 and maGSC SCC5 target cells was observed. These data indicate that the injection of the maGSCs SSC5 cells into the heart activates NK cells in RAG2^−/−^ mice and results in an increased killing not only of YAC-1 but also of maGSC SSC5 cells.

In parallel to the chromium release assays, the expression of ligands for activating (CD112, CD155, RAE-1) and inhibitory (H2K^b^, H2D^b^) NK receptors was determined by flow cytometry (Figure 2B). We analyzed the percentage of cells expressing these molecules as well as their mean fluorescence intensity. The expression of CD155, a ligand of DNAM-1, and RAE-1, a ligand of NKG2D, was higher in YAC-1 than in maGSC SSC5 cells. The major histocompatibility (MHC) class I molecules H2K^b^ and H2D^b^, which serve as ligands for mainly inhibitory Ly49 receptors, were only weakly expressed on maGSC SSC5 cells. YAC-1 cells are MHC class I-deficient cells of the H2^a^ haplotype and were therefore not tested for H2K^b^ and H2D^b^ expression. The maGSC SSC5 cells are also lacking H2K^q^ molecules, and none of the class I molecules (H2K^b^, H2D^b^, or H2K^q^) was decidedly induced by the stimulation with IFN-γ for 48 h (Figure 2C). These expression patterns contribute to the higher susceptibility of YAC-1 than maGSC SSC5 cells to killing by NK cells as demonstrated previously (20, 22).

### CsA Suppresses Teratoma Formation

Next, the experiments were done in the same way but the recipients were treated in addition with CsA (20 mg/kg/day, i.p.). Overall, the frequency of tumors (Table 1) in RAG2^−/−^ mice was lower than in RAG2^−/−^γc^−/−^ mice (*P* = 0.0567, χ^2^ test). The treatment with CsA led to a highly significant decrease of the teratoma frequency in RAG2^−/−^ and RAG2^−/−^γc^−/−^ mice in comparison to the untreated animals (*P* = 0.0019, χ^2^ test). In the RAG2^−/−^ mice treated with CsA, no teratomas were observed in the myocardium but in one mouse, a tumor was found on the heart (Table 1; Figure 1E). Only 60% of the CsA-treated RAG2^−/−^γc^−/−^ mice developed a teratoma upon intramyocardial transplantation of 5 × 10^5^ maGSC SSC5 cells (Table 1). The teratomas were also smaller with a mean length of 1.3 mm and a mean width of 0.8 mm than those in the CsA-untreated RAG2^−/−^γc^−/−^ mice (*P* < 0.001; Figure 1E). These results showed an unexpected but significant influence of CsA on the risk of teratoma formation after injection of maGSC SCC5 cells into the heart in two immunodeficient mouse lines.

**Figure 3:**
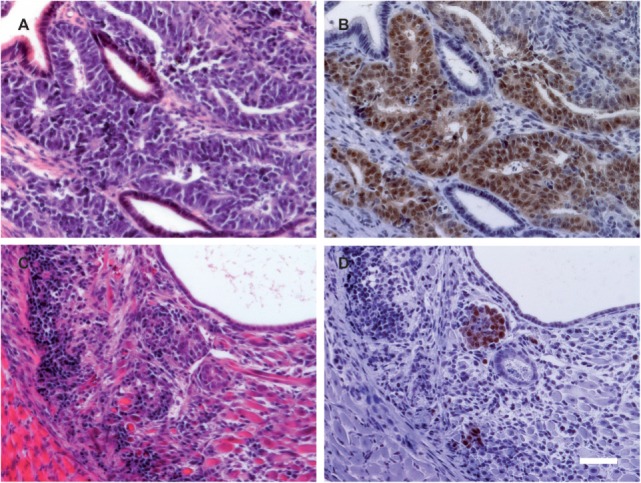
**The proportion of OCT4-positive cells is reduced in teratomas from cyclosporine A (CsA)-treated mice**. **(A,C)** H&E staining of representative teratomas grown in a RAG2^−/−^γc^−/−^ mouse not treated **(A)** or treated with CsA **(C)**. **(B,D)** On adjacent sections, OCT4 expression was determined by immunohistochemistry. The pictures are representative for the teratomas obtained in the RAG2^−/−^γc^−/−^ mice treated or not treated with CsA. Scale bar, 50 µm.

An immune histological analysis of the teratomas of the CsA-treated RAG2^−/−^γc^−/−^ mice indicated a reduced OCT4 expression in comparison to the non-CsA-treated group (Figure 3). Since OCT4 is a marker for undifferentiated PSCs, CsA seems to stimulate the differentiation of the transplanted undifferentiated cells.

### Cyclosporine Does Not Influence NK Cell Activity

**Figure 4:**
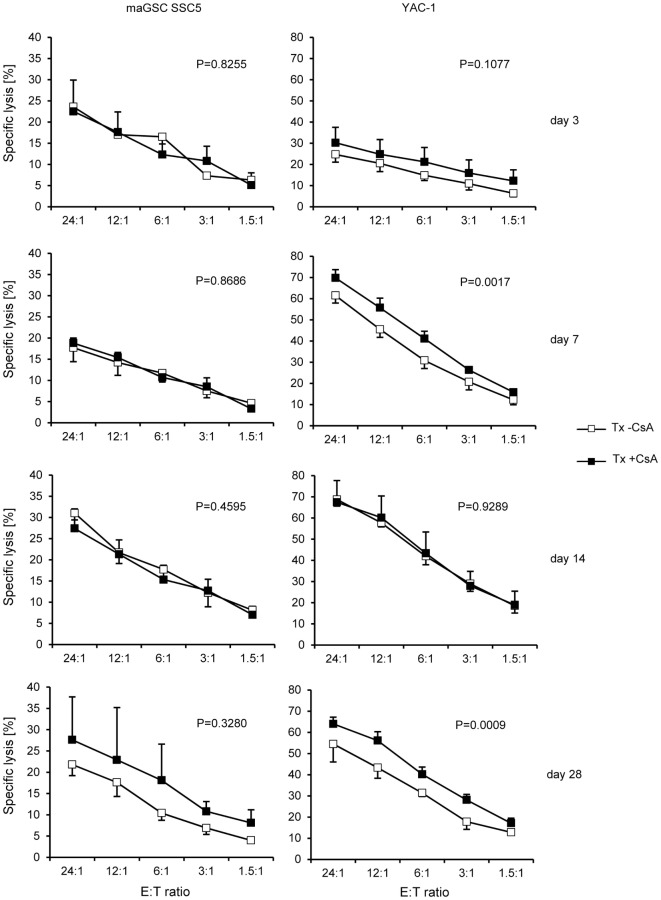
**Comparison of the killing of multipotent adult germline stem cell (maGSC) SSC5 and YAC-1 target cells by natural killer (NK) cells of RAG2^−/−^ mice treated or not treated with cyclosporine A (CsA)**. A summary of means of specific lysis and the SEM is shown as determined by ^51^Cr-release assays of maGSC SSC5 (left panels) and YAC-1 target cells (right panels) by NK cells. The NK cells were isolated from transplanted (Tx) RAG2^−/−^ mice receiving (+CsA) or not receiving (−CsA) the immunosuppressant CsA (20 mg/kg/day) at various days (3, 7, 14, or 28) after transplantation (*n* = 3 for each group). The results of the transplanted but not CsA-treated (−CsA) mice have been also displayed in Figure 3. The groups (Tx −CsA and Tx +CsA) have been compared by two-way ANOVA adjusted for E:T ratios and the respective *P* values are indicated.

Next, we analyzed the effect of CsA on the NK cell activity of RAG2^−/−^ mice. Overall, when summarizing all time points after operation, the activity of NK cells from CsA-treated mice against YAC-1 target cells was slightly increased (w/o CsA: 52% at the E:T ratio of 24:1, *n* = 12; with CsA: 59%, *n* = 14; *P* = 0.0083, two-way ANOVA adjusted for E:T ratios). However, the killing of maGSC SSC5 target cells was not different in mice treated or not treated with CsA (w/o CsA: 24% at the E:T ratio of 24:1, *n* = 12; with CsA: 25%, *n* = 14; *P* = 0.4472, two-way ANOVA adjusted for E:T ratios). A separate analysis of the NK cell cytotoxicity at the different time points after transplantation indicated no differences for maGSC SSC5 targets, but an increased killing of YAC-1 cells at day 7 (*P* = 0.0017) and day 28 after the transplantation (*P* = 0.0009, two-way ANOVA adjusted for E:T ratios) (Figure 4). The general pattern of NK cell activity after transplantation was not altered by the treatment with CsA. Thus, effects of CsA on the NK cell activity could not explain the effect of CsA on teratoma formation.

### NK Cell Cytotoxicity Is Increased in Wild-type Mice upon Operation

The results obtained in RAG2^−/−^ mice raised the questions whether the transplantation also activates NK cells in wild-type mice and whether NK cells become activated by the transplantation of maGSCs or in response to the surgery. Therefore, we determined the NK cell activity in C57BL/6 wild-type mice after intramyocardial transplantation of maGSC SSC5 cells. To differentiate effects of the operation (sham) from effects of the cell transplantation (Tx), we included sham-operated mice, which received an intramyocardial injection of 0.9% NaCl instead of cells. In addition to the treatment with CsA, we also included mice without the treatment with CsA. None of these mice developed a teratoma (Table 1). The NK cell activity was assayed 3, 7, and 14 days after transplantation since this was the most interesting phase with respect to cytotoxic NK cell activity in the RAG2^−/−^ mice.

**Figure 5:**
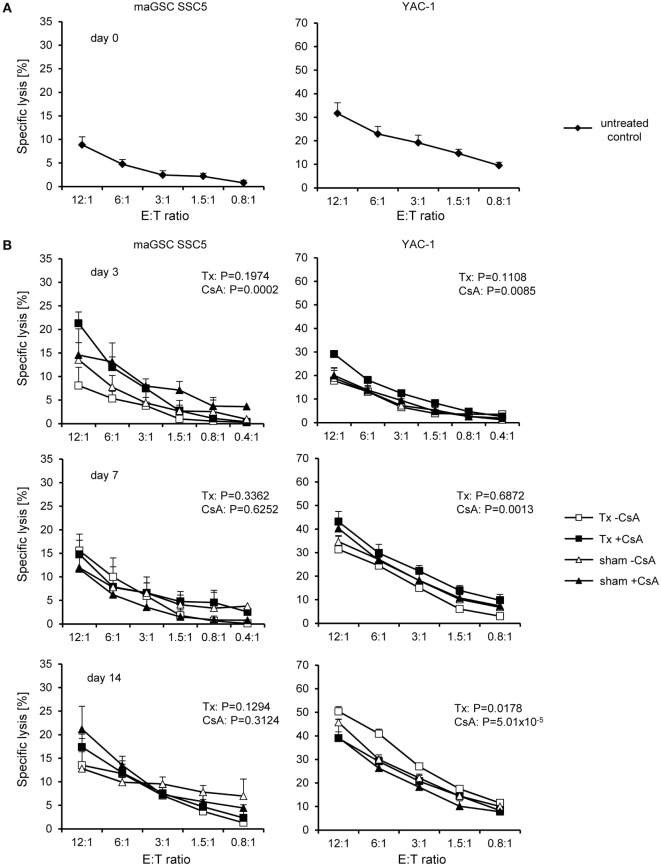
**Cytotoxic activity of natural killer (NK) cells at different time points after intramyocardial transplantation of multipotent adult germline stem cell (maGSC) SSC5 cells in C57BL/6 mice**. A summary of means of specific lysis and the SEM is shown as determined by ^51^Cr-release assays of maGSC SSC5 (left panels) and YAC-1 target cells (right panels) by NK cells. **(A)** The NK cells were isolated from C57BL/6 control mice (day 0) or **(B)** at various days (3, 7, or 14) after operation and directly assayed without prior stimulation *in vitro*. The NK cells were isolated from mice that received an intramyocardial transplantation of maGSC SSC5 cells (Tx) or sham-operated (sham) mice. The mice were treated (+CsA) or not treated (−CsA) with the immunosuppressant cyclosporine A (CsA) (20 mg/kg/day) (*n* = 3 per group and 4 for transplanted mice but not treated with CsA at day 7). The groups (−CsA and +CsA as well as sham and Tx) have been compared by two-way ANOVA adjusted for E:T ratios and the respective *P* values are indicated.

Mice that were not operated (day 0, untreated control) were included to determine the basal NK cell cytotoxicity (*n* = 3). Specific lysis of YAC-1 target cells was on average 32% at the E:T ratio of 12:1. In parallel, the specific lysis of the maGSC SSC5 cells was 9% (Figure 5A). Overall, the maGSCs were more resistant to killing by wild-type NK cells than YAC-1 cells (*P* = 2.85 × 10^−10^, two-way ANOVA adjusted for E:T ratios). When summarizing all data of the three time points after operation, which are shown in Figure 5B (days 3, 7, and 14), this difference of killing remained unchanged (*P* = 2.06 × 10^−43^, *n* = 38 per cell line). On average, 34% of YAC-1 and 15% of maGSC SSC5 cells were killed at the E:T ratio of 12:1. The target cell lysis, i.e., YAC-1 and maGSC SSC5 together, was affected by time (*P* = 1.31 × 10^−10^, two-way ANOVA adjusted for E:T ratios, day 3: *n* = 24, day 7: *n* = 28, day 14: *n* = 24). On average, specific lysis of both targets was 18% at day 3, 25% at day 7, and 30% at day 14 after the operation. However, killing was not different when a sham operation was compared to Tx (*P* = 0.6453, two-way ANOVA adjusted for E:T ratios, sham and Tx each *n* = 38). Furthermore, the killing of target cells was also not affected by the treatment with CsA (*P* = 0.2085, two-way ANOVA adjusted for E:T ratios, w/o CsA: *n* = 40; with CsA: *n* = 36). When YAC-1 and maGSC SSC5 target cells were analyzed separately, similar results were obtained, indicating a significant dependence of killing on the time after operation (*P* = 2.77 × 10^−28^ for YAC-1 and *P* = 0.0107 for maGSC SSC5 cells, two-way ANOVA adjusted for E:T ratios), but not on the type of operation (sham versus Tx) or on the treatment with CsA.

Similar to results obtained with NK cells of RAG2^−/−^ mice, the killing of YAC-1 cells by NK cells of wild-type mice was reduced at day 3 (*P* = 1.45 × 10^−9^), but increased at day 14 (*P* = 0.0212) and unchanged at day 7 (*P* = 0.6712, two-way ANOVA adjusted for E:T ratios). The cell transplantation, when compared to the sham operation, had no significant effect on the killing of YAC-1 targets, except at day 14 showing a slight increase from 42% (sham: *n* = 6) to 45% (Tx: *n* = 6) at the E:T ratio of 12:1 (*P* = 0.0178, two-way ANOVA adjusted for E:T ratios). This difference was also observed when the CsA-treated recipients were excluded from the analysis with 46% (sham, *n* = 3) versus 50% (Tx, *n* = 3) specific lysis of YAC-1 targets at the E:T ratio of 12:1 (*P* = 3.49 × 10^−05^, two-way ANOVA adjusted for E:T ratios). Effects of the treatment with CsA were rather complex for the various time points after transplantation. At day 3, the killing of maGSC SSC5 (*P* = 0.0002) and YAC-1 cells (*P* = 0.0085, two-way ANOVA adjusted for E:T ratios) was higher by NK cells of mice treated with CsA than untreated mice. The same was observed at day 7 for YAC-1 targets (*P* = 0.0013, two-way ANOVA adjusted for E:T ratios). However, at day 14, the cytotoxic NK cell activity of mice treated with CsA against YAC-1 cells was reduced (*P* = 5.01 × 10^−5^, two-way ANOVA adjusted for E:T ratios). Thus, the effects of the stem cell transplantation on cytotoxic NK cell activity were present also in wild-type mice mainly due to the operation but not the transplanted cells. Notably, CsA had little effects on NK cell cytotoxicity when averaged over the complete time of treatment.

### Inflammatory Response to Heart Surgery

**Figure 6:**
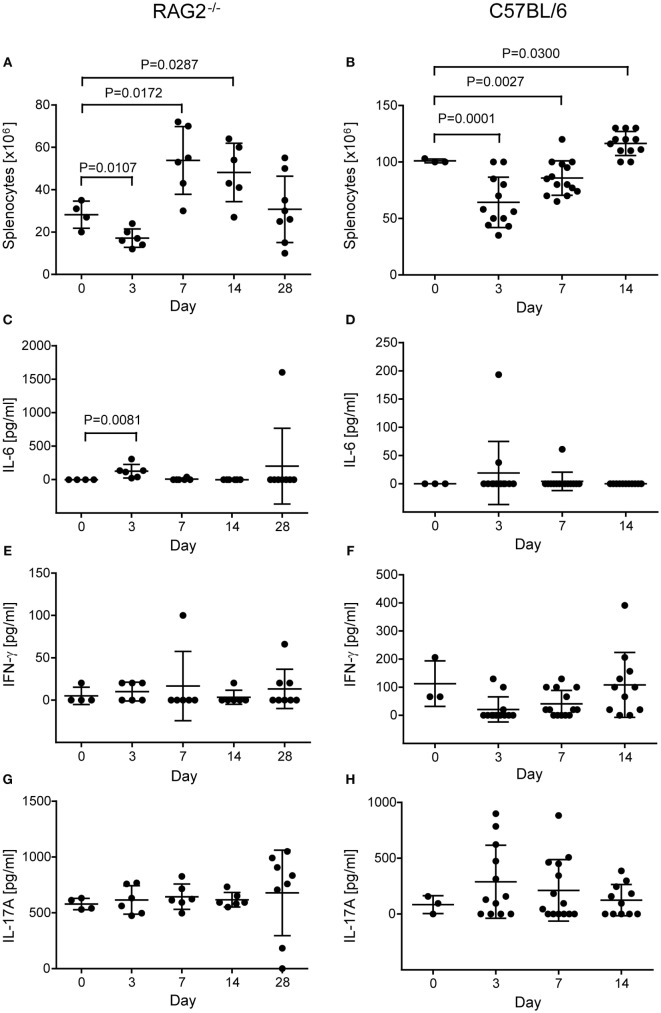
**Number of splenocytes and concentration of cytokines in non-operated control and transplanted RAG2^−/−^ (A,C,E,G) and C57BL/6 mice (B,D,F,H)**. The number of splenocytes was counted after lysis of erythrocytes and before isolation of natural killer cells in RAG2^−/−^ **(A)** and C57BL/6 **(B)** mice not operated (day 0) and at various days (3, 7, 14, and for RAG2^−/−^ mice 28) after transplantation. Differences in splenocyte numbers between not-operated controls and transplanted mice were compared by *t*-tests and the significant *P* values are displayed. The concentrations of cytokines IL-6 [**(C)** RAG2^−/−^; **(D)** C57BL/6], interferon-γ [**(E)** RAG2^−/−^; **(F)** C57BL/6], and IL-17A [**(G)** RAG2^−/−^; **(H)** C57BL/6] were measured by enzyme-linked immunosorbent assay in the sera of the mice. The mean + SD are indicated. The significant difference in IL-6 concentration between controls (non-operated) and RAG2^−/−^ mice at day 3 after operation was calculated by the non-parametric *U* test.

When we determined the number of splenocytes in RAG2^−/−^ mice before isolation of NK cells, we realized a dependency on the time point after operation (*P* = 0.0003, two-way ANOVA adjusted for treatment with CsA). Compared to non-operated control mice, cell numbers were decreased at day 3 (*P* = 0.0107) but increased at day 7 (*P* = 0.0172) and 14 (*P* = 0.0287, Student’s *t-*tests) after the operation (Figure 6A). This likely indicates an early recruitment of immune cells from the spleen to a site of inflammation, such as the operation wound of the heart, and a lymphocytosis at later stages. The treatment with CsA overall increased the number of splenocytes (*P* = 0.0304, two-way ANOVA adjusted for the time after operation), but at no single time point the difference reached statistical significance (Figure S1A in Supplementary Material).

To further analyze the postoperative inflammation, we determined several cytokines in the serum of the mice. The pro-inflammatory cytokines TNF-α and IL-1b, and the Th_2_ cytokine IL-4 were not detectable systemically (data not shown). The IL-6 concentration in serum was dependent on the time after operation (*P* = 0.0013; *H* test) and was increased at day 3 compared to controls (*P* = 0.0081, *U* test), indicating an early but transient inflammatory response after the operation (Figure 6C). In addition, IL-6 was high (1.602 pg/ml) in a single mouse at day 28, in which a teratoma was developed (Figure 6C). The systemic concentration of the Th_1_ cytokine IFN-γ, which can be produced, e.g., by activated NK cells, did not show a dependency on the time after operation (*P* = 0.6422, *H* test) and was not increased at any single time point when compared to control mice (Figure 6E). Similarly, the Th_17_ cytokine IL-17A was not increased in a time-dependent manner after operation (*P* = 0.4356, *H* tests) (Figure 6G). Moreover, none of the cytokine concentrations was dependent on the treatment with CsA except IL-17A, which was overall slightly increased in mice treated with CsA (542 ± 190 versus 728 ± 213 pg/ml, *P* = 0.0080, H test) (Figure S1D in Supplementary Material). Only at day 7 after transplantation, the difference reached statistical significance for a single time point (*P* = 0.0495, *U* test). However, the relevance of this difference remains unclear.

Similarly, we determined the number of splenocytes before isolation of NK cells in C57BL/6 wild-type mice and found again a dependency on the time point after operation (*P* = 3.00 × 10^−8^, one-way ANOVA) and the *post hoc* test indicated a difference between the three time points (days 3, 7, and 14) (Figure 6B). The number of splenocytes remained significantly different when adjusted for the type of operation (sham versus Tx: *P* = 4.07 × 10^−8^) or the treatment with CsA (*P* = 1.23 × 10^−9^). Overall, neither the type of operation (*P* = 0.1937) nor the treatment with CsA (*P* = 0.1167) had a significant effect on the number of splenocytes. Compared to untreated control mice (day 0), the number of splenocytes was reduced at day 3 (*P* = 0.0001) and 7 (*P* = 0.0027) but increased at day 14 (*P* = 0.0300, Student’s *t-*tests) after operation (Figure 6B). This pattern was similar to the results obtained in RAG2^−/−^ mice (Figure 6A). At day 3, the number of splenocytes was affected by the CsA treatment (*P* = 0.0070, two-way ANOVA adjusted for the type of operation) (Figure S2A in Supplementary Material). It was reduced in mice treated with CsA when compared to mice not receiving CsA (*P* = 0.0031, Student’s *t-*test). At day 7, the number of splenocytes was affected by the type of operation (*P* = 0.0474, two-way ANOVA adjusted for CsA treatment) (Figure S2A in Supplementary Material) and higher in Tx mice compared to sham-operated mice (*P* = 0.0303, Student’s *t-* test).

The systemic concentration of IL-6 was increased only in three C57BL/6 wild-type mice after operation (Figure 6D) and the concentrations of IFN-γ and IL-17A in the sera were not significantly dependent on the time point after operation (Figures 6F,H). IL-4 was not detected in the sera of these mice. The type of operation or the treatment with CsA had no effect on IL-6 (Figure S2B in Supplementary Material) or IFN-γ (Figure 2C) concentration in the serum. At day 3, the IL-17A concentrations were higher in sham-operated than in Tx mice (*P* = 0.0159, *H* test) (Figure S2D in Supplementary Material). However, in view of the rather high variation of the concentration of this cytokine also at the other time points, the relevance of this finding remains questionable.

In summary, we found indications for a systemic inflammatory response in the operated RAG2^−/−^ mice and to a lesser extend in C57BL/6 wild-type mice. Although no clear polarization toward a Th_1_ response was found, our results showed that NK cells became activated in transplanted and sham-operated mice. This activation of NK cells could reduce the risk of teratomas in recipients receiving grafts of *in vitro* differentiated cells that might be contaminated with traces of PSCs.

## Discussion

Pluripotent stem cells hold great promises for regenerative medicine as a source of cells and tissues for treatment of diseases such as macular degeneration or heart failure (33–35). The type of PSCs best suited for therapeutic purposes is currently debated (36). The use of ESCs is associated with ethical problems and they can only be used in allogenic transplant settings. In contrast, autologous iPSCs can be generated from patients, but they might be compromised in their genetic integrity by preexisting somatic mutations or mutations occurring during the reprograming process (11). The individual patient-specific iPSC lines would need to undergo complex procedures for quality control before being used as a source for autologous grafts, which are expected to be time consuming and expensive. For these reasons, only allogeneic transplants might be feasible for urgent and frequent medical indications and this would require the definition of an appropriate immunosuppressive therapy for the recipients (37). Besides the rejection of grafts, the formation of teratomas by residual PSCs within a graft is a major risk associated with the transplantation of PSC-derived cells or tissues (10). However, this risk would be increased in autologous transplant settings or in immunosuppressed recipients of allogeneic grafts (29, 38).

In this study, we have investigated the intramyocardial transplantation of murine maGSCs, which are an alternative PSC type that can be obtained by ethically acceptable procedures from adult SSCs without the need of genetic manipulation.

First, we addressed the question whether the site of transplantation affects the risk of teratomas arising from maGSCs. Our previous studies showed that undifferentiated maGSC SSC5 cells formed teratomas in immunodeficient mice after subcutaneous injection (7, 21). Moreover, further maGSC lines from other mouse strains were also capable to form teratomas in immunodeficient recipients after subcutaneous injection (22). In partially allogeneic C57BL/6 wild-type mice immunosuppressed with CsA, however, no teratomas were found within 4 weeks after transplantation of maGSCs SCC5 cells into the heart (8). We now show that teratomas were formed regularly also in the heart of immunodeficient recipients after intramyocardial injection of maGSC SSC5 cells. Moreover, additional teratomas at distant sites occurred in 75% of the RAG2^−/−^γc^−/−^ recipients. The microenvironment of the heart has therefore no specific cardio-instructive potential for maGSCs. Mouse ESCs have been reported to differentiate upon transplantation in infarcted myocardium into cardiomyocytes, vascular smooth muscle, and endothelial cells and to improve cardiac structure and function (39). However, others studies emphasized that ESCs need to be differentiated before transplantation to avoid teratoma formation in the heart (40). On the other hand, the maGSCs were rejected in our study also after subcutaneous injection into immunocompetent C57BL/6 mice. Since, the maGSC SSC5 line was derived from a mouse with a mixed background (7), a rejection of the partially allogenic maGSCs by the immunocompetent C57BL/6 recipients had to be expected. However, recipients treated with CsA for immunosuppression also rejected the cells. This demonstrates that the risk of rejection does not differ after subcutaneous and intramyocardial transplantation and it suggests that non-T cells mediate the rejection of the maGSCs because T cells are the main targets of CsA. We have previously shown that NK cells can contribute to the rejection of PSCs, including ESCs, iPSCs, and maGSCs, after subcutaneous injection although they were not sufficient to prevent teratoma growth completely (20, 22, 29). After intramyocardial transplantation of maGSCs, we found now a reduced frequency and a reduced size of teratomas in T and B-cell-deficient RAG2^−/−^ recipients compared to additionally NK cell-deficient RAG2^−/−^γc^−/−^ mice. This indicates that NK cells impair the growth of teratomas not only in the subcutaneous tissue but also in the heart.

Initially, the effect of NK cells on PSCs has been debated and some studies reported resistance or very low susceptibility of the stem cells (41, 42), while others and we found PSCs to be highly susceptible to killing by NK cells (20, 22, 28). The conflicting results appear to reflect mainly differences in the activation status of NK cells. We have recently shown that human iPSCs are largely resistant to resting allogenic as well as syngeneic NK cells but were readily killed by IL-2-activated NK cells (30). Moreover, we demonstrated that activation of murine NK cells *in vivo* by poly (I:C) impaired teratoma growth (22). The killing of murine PSCs was mediated in part by the activating NK receptor NKG2D (20, 22, 28), while killing of human iPSCs were more dependent on the activating NK receptor DNAM-1 (30). The maGSC SSC5 expressed some ligands for NKG2D and DNAM-1 but only low levels of MHC class I molecules that function as ligands for inhibitory NK receptors. This expression pattern is similar to other PSCs (21, 22), and it could contribute to the susceptibility of the maGSCs to killing by activated NK cells. Exposure to IFN-γ for 48 h was not sufficient to induce the expression of MHC class I molecules on the maGSCs suggesting that a pro-inflammatory milieu *in vivo* would also not render the undifferentiated cells resistant to NK cells.

Upon differentiation, however, the expression MHC class I molecules increases on most cell types rendering them less susceptible to NK cells (22). *In vivo*, injected PSCs can escape rejection by NK cells by differentiation into the various cell types present in teratomas. This, however, is expected to increase the risk of rejection of allografts by alloreactive CTL and other mechanisms of the adaptive immune system. Therefore, NK cell can frequently not prevent teratoma growth completely upon injection of larger amounts of PSCs, whereas no teratomas occur in immunocompetent recipients receiving allogeneic PSCs.

Natural killer cells are an interesting player in the transplantation of PSC-derived grafts because activated NK cells can target even autologous PSCs (30), while differentiated cells are normally resistant at least to autologous NK cells. NK cells might therefore be able to target specifically residual PSCs in PSC-derived grafts. Thereby, they potentially increase the safety of PSC-derived grafts (29), if their activity is not impaired by the immunosuppressive treatment given to the recipients. In this study, we have shown that NK cells become activated in RAG2^−/−^ and in wild-type recipients upon transplantation. The *in vivo*-activated NK cells killed the NK cell target cell line YAC-1 and, although less efficiently, the maGSCs. Notably, the maGSCs were largely resistant to freshly isolated NK cells from untreated control mice. The highest activity was observed 2 weeks after the transplantation. One week after the transplantation, we observed a systemic inflammatory response with a slightly increased serum concentration of IL-6 and a lymphocytosis in the spleens of RAG2^−/−^ mice. In wild-type mice, the lymphocytosis occurred later, 2 weeks after transplantation, and the IL-6 response was restricted to individual mice. No clear systemic polarization toward a Th_1_, Th_2_, or Th_17_ cytokine profile was observed at the systemic level in the serum of the recipients. However, in the cause of the inflammatory response, the NK cell activity increased in RAG2^−/−^ and wild-type recipients. Experiments in wild-type recipients furthermore demonstrated that the sham operation procedure was sufficient to activate the NK cells. It remains to be investigated whether a less traumatic transplantation procedure than open-chest surgery, such as ultrasound-guided injection of cells into the heart, would also activate the NK cell system. If not, the risk of teratoma growth could be higher in NK cell competent recipients than in the experiments reported here. Importantly, the activation of NK cells was not impaired by treatment of the recipients with CsA. This could explain why CsA is not sufficient to allow for an engraftment of the partially allogeneic maGSCs in C57BL/6 recipients. This is in line with our previous results obtained with an ESC line, which has been usually rejected in CsA-treated allogeneic recipients (20). However, effects of CsA and further immunosuppressive drugs on the peripheral NK cell repertoire and NK cell activity have been observed in transplant patients (43) and need to be further considered when developing allogeneic PSC-based transplant therapies. Recently, teratocarcinomas have been observed after allogeneic transplantation of iPSC-derived cardiac tissue sheets in syngeneic C57BL/6 and with tacrolimus-treated allogeneic BALB/c mice but not in the immunocompetent BALB/c recipients (44). The authors presented some evidence that the NK cell activity might have been suppressed by tacrolimus, and therefore, tacrolimus could be associated with a higher tumor risk than CsA after transplantation of PSC-derived grafts.

Notably, we observed in our study also that the CsA treatment impaired the teratoma growth in immunodeficient recipients. In teratomas from CsA-treated RAG2^−/−^γc^−/−^ mice, we observed a reduced expression of OCT4, which is an essential transcription factor in PSCs. This suggests that CsA could inhibit the survival or proliferation of PSCs or promote their differentiation into non-proliferating tissues. Interestingly, it has been reported that CsA can indeed enhance the cardiac differentiation of mouse ESCs (45) and mouse and human iPSCs (46). Moreover, a reduced proliferation of other stem cell types including neural stem cells and mesenchymal stem cells has been observed [reviewed in Ref. (47)]. These results indicate that direct effects of immunosuppressive drugs on PSCs and PSC-derived grafts need to be considered. In future studies, different immunosuppressive or immunomodulatory treatments (48–51) need to be compared to define the best regimen allowing for engraftment of allogeneic PSC-derived transplants without increasing the risk of tumorigenicity.

## Author Contributions

KG and RD designed the study; DH, DK, and LE acquired data; DH, DK, RD, SM, and KG analyzed and interpreted data; RD, DH, and KG wrote the manuscript; DH, DK, LE, SM, RD, and KG approved the final version of the manuscript.

## Conflict of Interest Statement

The authors declare that the research was conducted in the absence of any commercial or financial relationships that could be construed as a potential conflict of interest.
