# Supplementary material for: The Tumorigenicity of Multipotent Adult Germline Stem Cells Transplanted into the Heart Is Affected by Natural Killer Cells and by Cyclosporine A Independent of Its Immunosuppressive Effects
Source: Front Immunol. 2017 Feb 6;8:67. doi: 10.3389/fimmu.2017.00067 (PMC5292627; doi:10.3389/fimmu.2017.00067)
Supplement: Supplementary file 1 [file Data_Sheet_1.PDF]

Supplementary Material

The tumorigenicity of multipotent adult germline stem cells (maGSCs) transplanted into the heart is affected by natural killer cells and by cyclosporine A independent of its immunosuppressive effects

Daniela Hübscher, Diana Kaiser, Leslie Elsner, Sebastian Monecke, Ralf Dressel, Kaomei Guan

- Supplementary Table 1
- Supplementary Figure 1
- Supplementary Figure 2

Supplementary Table 1. Antibodies and isotype controls used for flow cytometry

**Antigen**	**Isotype**	**Clone**	**Label**	**Supplier**
CD3	rat IgG_{2b}	17A2	FITC	BioLegend, Fell, Germany
CD49b	rat IgM	DX5	PE	BioLegend, Fell, Germany
CD112	rat IgG_{2a}	502-57	-	Santa Cruz, Heidelberg, Germany
CD155	rat IgG_{2a}	TX56	-	BioLegend, Fell, Germany
RAE-1	rat IgG_{2a}	186107	-	R&D Systems, Wiesbaden, Germany
$H2K^b$	mouse IgG_{2a}	AF6-885	PE	BioLegend, Fell, Germany
$H2D^b$	mouse IgG_{2b}	KH95	PE	BioLegend, Fell, Germany
$H2K^q$	mouse IgG_{2a}	KH114	FITC	BioLegend, Fell, Germany
mouse IgG	goat IgG	polyclonal (155-095-062)	FITC	Jackson Laboratories, via Dianova, Hamburg, Germany
rat IgG	goat IgG	polyclonal (112-095-062)	FITC	Jackson Laboratories, via Dianova, Hamburg, Germany
-	rat IgM	RTK2118	PE	BioLegend, Fell, Germany
-	rat IgG_{2b}	RTK4530	FITC	BioLegend, Fell, Germany
-	mouse IgG_{2a}	713	FITC	Immunotools, Friesoythe, Germany
-	mouse IgG_{2a}	MOPC-173	PE	BioLegend, Fell, Germany
-	mouse IgG_{2b}	MPC-11	PE	BioLegend, Fell, Germany

The following abbreviations are used: FITC, fluorescein isothiocyanate, and PE, phycoerythrin.

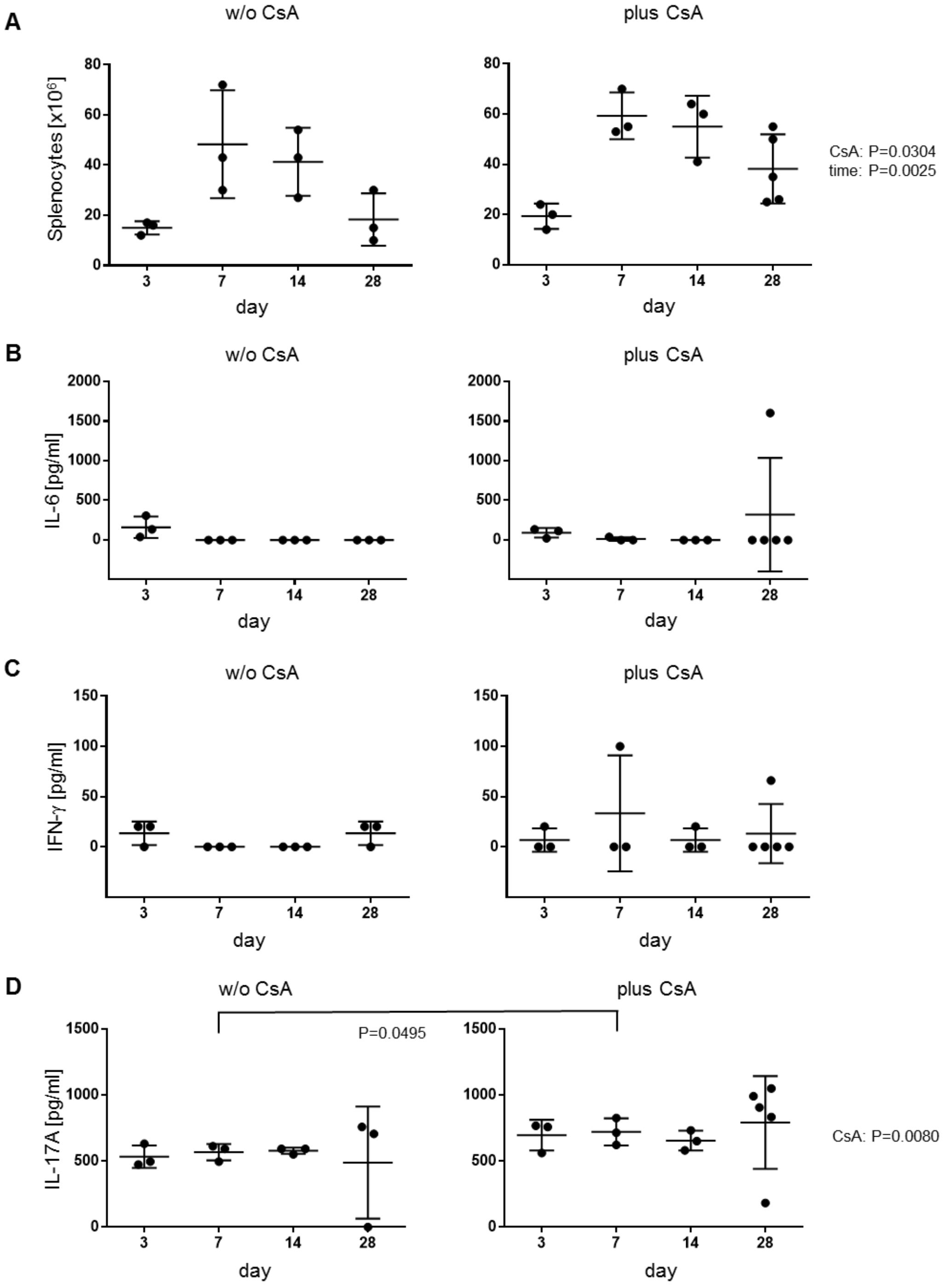

Supplementary Figure 1: Number of splenocytes and concentration of cytokines in transplanted RAG2$^{-/-}$ mice receiving or not receiving CsA. (A) The number of splenocytes was counted after lysis of erythrocytes and before isolation of NK cells at days 3, 7, 14, and 28 after transplantation. Effects of CsA-treatment and time after transplantation on splenocyte numbers were analyzed by 2-way ANOVA and the respective P values are displayed. **(B, C, D)** The concentrations of cytokines (IL-6, IFN-γ, and IL-17A) were measured by ELISA in the sera of the mice. The means ± SD are indicated. Effects of CsA-treatment were analyzed by the non-parametric H test and significant P values are displayed. Post hoc comparisons for single days indicated a difference of IL-17 concentrations depending on CsA-treatment at day 7 after operation (P=0.0495; U test).

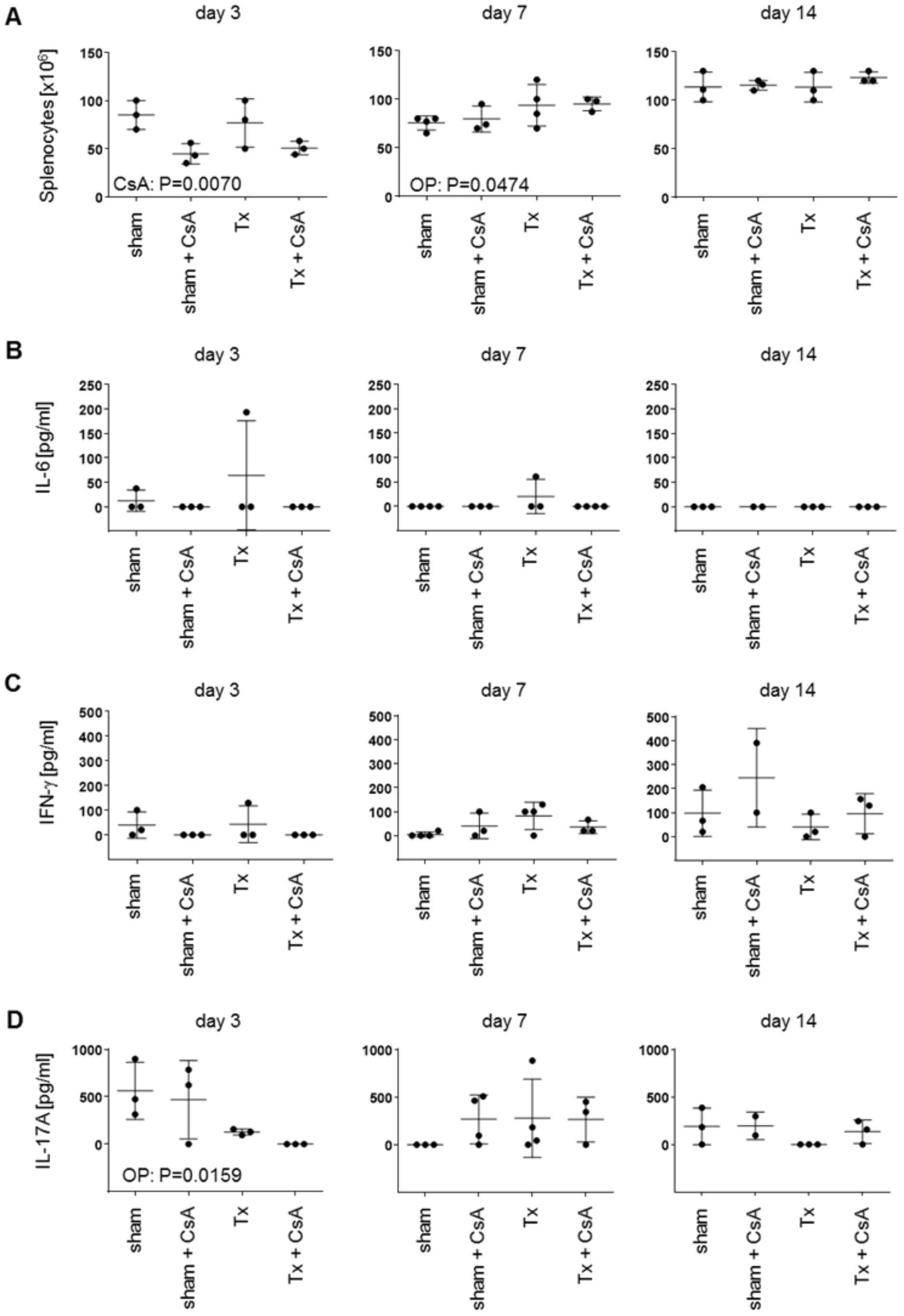

Supplementary Figure 2: Number of splenocytes and concentration of cytokines in transplanted C57BL/6 mice receiving or not receiving CsA. (A) The number of splenocytes was counted after lysis of erythrocytes and before isolation of NK cells at days 3, 7, and 14 after transplantation. Effects of CsA-treatment and type of operation (OP, sham versus Tx) were analyzed by 2-way ANOVA and the respective P values are displayed. **(B, C, D)** The concentrations of cytokines (IL-6, IFN-γ, and IL-17A) were measured by ELISA in the sera of the mice. The means ± SD are indicated. Effects of CsA-treatment and type of operation were analyzed by the non-parametric H test and significant P values are displayed.
